# Epigallocatechin gallate inhibits SNARE‐dependent membrane fusion by blocking *trans*‐SNARE assembly

**DOI:** 10.1002/2211-5463.13488

**Published:** 2022-09-26

**Authors:** Min Zhu, Han Xu, Yuting Jiang, Haijia Yu, Yinghui Liu

**Affiliations:** ^1^ Jiangsu Key Laboratory for Molecular and Medical Biotechnology, College of Life Sciences Nanjing Normal University China

**Keywords:** EGCG, exocytosis, insulin secretion, membrane fusion, SNARE, synaptotagmin

## Abstract

Insulin secretion is a signal‐triggered process that requires membrane fusion between the secretory granules and plasma membrane in pancreatic β cells. The exocytosis of insulin is mediated by target‐soluble *N*‐ethylmaleimide sensitive factor attachment protein receptors (SNAREs) on the plasma membrane and vesicle‐SNAREs on the vesicles, which assemble into a quaternary *trans*‐SNARE complex to initiate the fusion. Expression of fusion proteins is reduced in the islets of patients with type II diabetes, indicating that SNARE‐mediated fusion defect is closely related to insulin‐based metabolic diseases. Previous studies have suggested that epigallocatechin gallate (EGCG) has an inhibitory effect on membrane fusion. In the present study, we performed *in vitro* reconstitution assays to unravel the molecular mechanisms of EGCG in SNARE‐mediated insulin secretory vesicle fusion. Our data show that EGCG efficiently inhibits insulin secretory SNARE‐mediated membrane fusion. Mechanistic studies indicated that EGCG blocks the formation of the *trans*‐SNARE complex. Furthermore, calcium/synaptotagmin‐7‐stimulated fusion kinetics were largely reduced by EGCG, confirming that it is a potential regulator of SNARE‐dependent insulin secretion. Our findings suggest that the *trans*‐SNARE complex might be a promising target for controlling SNARE‐dependent vesicle fusion.

## Graphical abstract

Insulin secretion is mediated by soluble *N*‐ethylmaleimide sensitive factor attachment protein receptor (SNARE)‐dependent membrane fusion. In the present study, we show that epigallocatechin gallate, a polyphenol compound, efficiently inhibited the insulin secretory SNARE‐mediated membrane fusion by blocking the formation of the *trans*‐SNARE complex. Our findings suggest that the *trans*‐SNARE complex might be a promising target for controlling vesicle fusion during exocytosis.

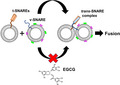

**Abbreviations**
ANOVA: analysis of variance
CD: cytoplasmic domain
DPPE: 1,2‐dipalmitoyl phosphatidylethanolamine
EGCG: epigallocatechin gallate
FRET: Förster resonance energy transfer
GTE: green tea extract
NBD: *N*‐(7‐nitro‐2,1,3‐benzoxadiazole‐4‐yl)
PM: plasma membrane
POPC: 1‐palmitoyl‐2‐oleoyl‐*sn*‐glycero‐3‐phosphocholine
POPE: 1‐palmitoyl‐2‐oleoyl‐*sn*‐glycero‐3‐phosphoethanolamine
POPS: 1‐palmitoyl‐2‐oleoyl‐*sn*‐glycero‐3‐phosphoserine
SNARE: soluble *N*‐ethylmaleimide sensitive factor attachment protein receptor
Syt7: synaptotagmin‐7
t‐SNARE: target‐SNARE
v‐SNARE: vesicle‐SNARE

Exocytosis is a fundamental biological process that transports cargoes encapsulated in the vesicles to the extracellular matrix [1, 2]. The imbalance of this process may cause severe disorders. It has been well established that exocytosis is driven by the soluble *N*‐ethylmaleimide‐sensitive factor attachment protein receptors (SNAREs) and their regulators [3, 4]. The target (t‐) SNAREs and vesicle (v‐) SNARE zipper from the N‐ to C‐terminus to form a four‐helix *trans*‐SNARE complex that overcomes the free energy barrier and initiates the fusion [5, 6, 7, 8]. The SNARE zippering is further controlled by regulatory factors or signaling molecules, corresponding to the spatially and temporally precise requirements of regulated exocytosis [9, 10, 11, 12, 13, 14].

Insulin, a peptide hormone, plays an essential role in cellular glucose uptake and usage. The defect of insulin secretion leads to a variety of metabolic diseases. Insulin secretion is a glucose‐stimulated process that requires membrane fusion between the secretory granules and plasma membrane (PM) in pancreatic β cells [1, 15]. In response to an increase in glucose concentration, insulin secretion exhibits a biphasic secretory reaction with a first peak followed by a lower sustained second phase [16, 17]. The exocytosis of insulin is primarily mediated by t‐SNAREs syntaxin‐1/SNAP‐25 on the PM and v‐SNARE VAMP2 on the vesicles [18, 19, 20, 21], which assemble into a quaternary *trans*‐SNARE complex to open the fusion pore [22]. Furthermore, another t‐SNARE pair syntaxin‐4/SNAP‐23 was also involved in insulin secretion [23, 24]. Glucose signals induce calcium influx from voltage‐dependent Ca^2+^ channels and synaptotagmin‐7 (Syt7) then couples with calcium to trigger SNARE‐dependent membrane fusion [25, 26]. Earlier reports found that the Syt7‐knockout mice showed a severe defect of glucose‐stimulated insulin secretion [27]. A reduced expression pattern of membrane fusion proteins was found in type II diabetes patients' islets, indicating that the fusion defect is closely related to insulin‐based metabolic diseases [28, 29].

Some regulated exocytosis, such as the degranulation of mast cells or synaptic exocytosis, could be regulated by phenolic compounds [30, 31, 32, 33, 34]. Yang et al. [34] reported that delphinidin and cyanidin could inhibit the N‐terminal SNARE zippering, whereas myricetin intercalated into the hydrophobic layers near the middle of the SNARE complex to block the SNARE zippering. Multiple studies reported that polyphenols, such as tea polyphenols and grape polyphenols, modulate blood glucose and alleviate type II diabetes by facilitating glucose uptake or improving insulin sensitivity [35, 36, 37, 38]. Epigallocatechin gallate (EGCG), a polyphenol substance accounting for 50–70% of green tea extract (GTE), is the main active ingredient of green tea [38, 39, 40]. It is a typical flavone‐3‐ol phenolic compound with eight free hydroxyl groups (Fig. 1A). Evidence obtained from cell culture and animal studies suggests that EGCG has beneficial effects on improving multiple diseases, including diabetes and cardiovascular diseases [38, 41, 42, 43]. Previous studies suggested EGCG has an inhibitory effect on membrane fusion, although its activity is less potent than other polyphenolic reagents such as myricetin, delphinidin or cyanidin [34].

**Fig. 1:**
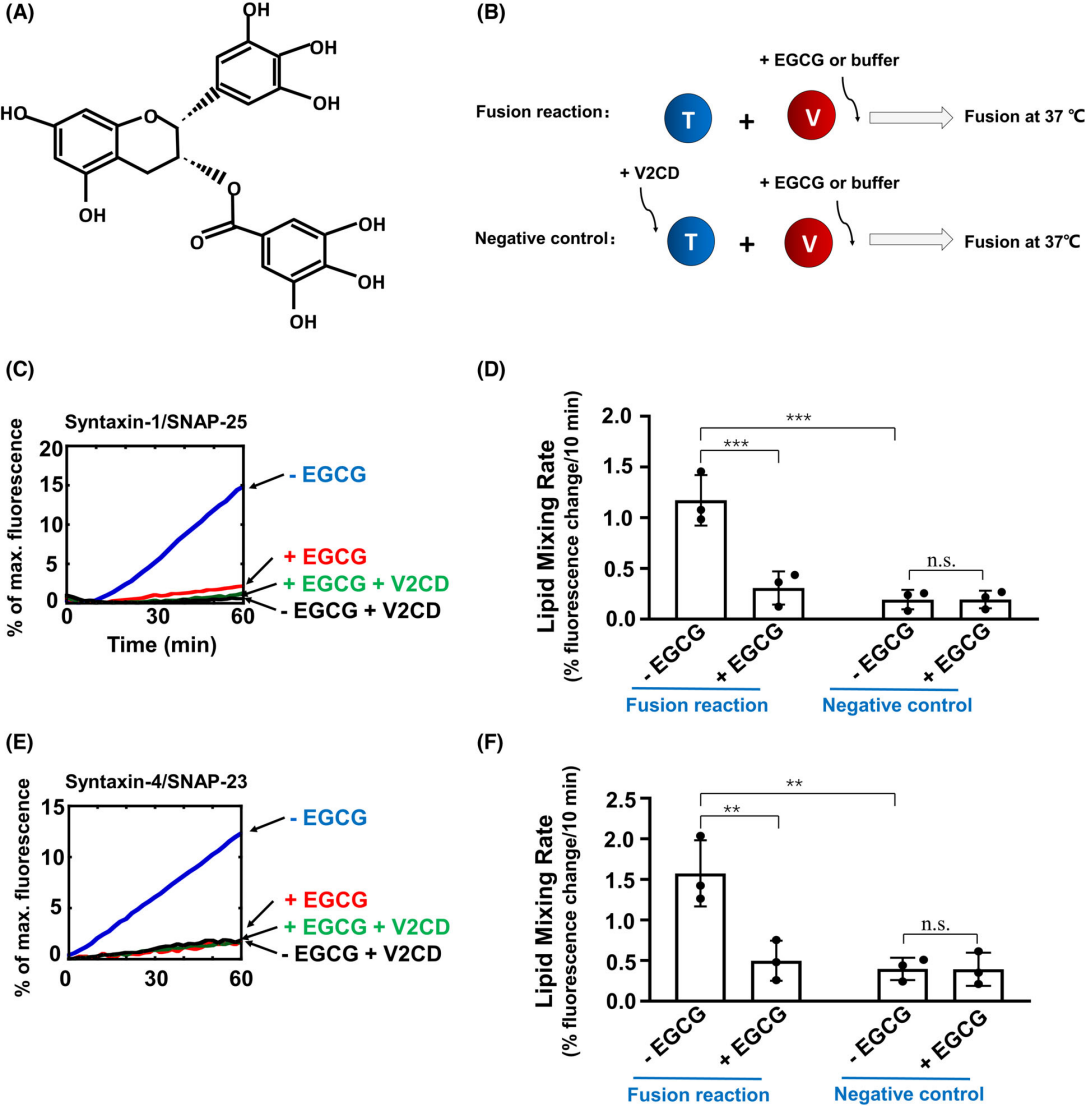
EGCG inhibits insulin secretory SNARE‐mediated lipid mixing reaction. (A) Chemical structure of EGCG. (B) Illustrations of the liposome fusion procedures. The t‐SNARE liposomes were reconstituted with syntaxin‐1/SNAP‐25, whereas the v‐SNARE liposomes contained VAMP2. (C) Fusion of the reconstituted proteoliposomes in the absence or presence of 10 μm EGCG. Negative controls: 20 μm V2CD was added at the beginning of the reactions. Each fusion reaction contained 5 μm t‐SNAREs and 1.5 μm v‐SNARE. The fusion reactions were measured using a FRET‐based lipid mixing assay. (D) Initial lipid mixing rates of the liposome fusion reactions shown in (C). Data are presented as a percentage of fluorescence change per 10 min. Error bars indicate the SD. Data are presented as the mean ± SD (*n* = 3 independent replicates). *P* values were calculated using two‐way ANOVA with Tukey's multiple comparisons test. n.s., *P* > 0.05; ***P < 0.001. (E) Fusion of the reconstituted proteoliposomes in the absence or presence of 10 μm EGCG. The t‐SNARE liposomes were reconstituted with syntaxin‐4 and SNAP‐23, whereas the v‐SNARE liposomes were prepared using VAMP2. Negative controls: 20 μm V2CD was added at the beginning of the reactions. Each fusion reaction contained 5 μm t‐SNAREs and 1.5 μm v‐SNARE. The fusion reactions were measured using a FRET‐based lipid mixing assay. (F) Initial lipid mixing rates of the liposome fusion reactions shown in (E). Data are presented as a percentage of fluorescence change per 10 min. Error bars indicate the SD. Data are presented as the mean ± SD (*n* = 3 independent replicates). *P* values were calculated using two‐way ANOVA with Tukey's multiple comparisons test. n.s., *P* > 0.05; **P < 0.01.

Here, we unravel the molecular mechanisms of EGCG in SNARE‐mediated insulin secretory vesicle fusion by *in vitro* reconstitution assays. We observed that EGCG efficiently inhibited the insulin secretory SNARE‐mediated membrane fusion. The inhibition of membrane fusion by EGCG is compatible with multiple v‐SNARE isoforms, including VAMP2, VAMP3 and VAMP8, which all support insulin secretion *in vivo* [44, 45, 46]. Further studies indicated that EGCG retards membrane fusion by blocking the formation of the *trans*‐SNARE complex. Interestingly, EGCG could further inhibit Ca^2+^/Syt7‐stimulated fusion when we reconstitute the Syt7 with the v‐SNARE liposomes, confirming EGCG is a negative regulator in SNARE‐dependent insulin secretion.

## Results

### EGCG dose‐dependently inhibits SNARE‐driven membrane fusion

We first explored how EGCG affects SNARE‐dependent membrane fusion. The insulin secretory SNAREs were reconstituted into a defined fusion system in which the v‐ and t‐SNAREs were anchored in separate populations of proteoliposomes (Fig. 1B). In a Förster resonance energy transfer (FRET)‐based lipid mixing assay, v‐ and t‐SNAREs drove an efficient level of lipid mixing [47, 48]. The fusion activity of SNAREs was entirely blocked by the cytoplasmic domain of VAMP2 (V2CD), a dominant‐negative inhibitor of *trans*‐SNARE assembly, suggesting that the change of lipid mixing is caused by the SNARE complex [47, 48]. When EGCG was included, the SNARE‐mediated lipid mixing was reduced to a background level comparable to that in the negative control reaction with V2CD (Fig. 1C,D). Interestingly, EGCG also inhibited syntaxin‐4/SNAP‐23, another t‐SNARE pair involved in insulin secretion, and VAMP2‐driven membrane fusion (Fig. 1E,F).

**Fig. 2:**
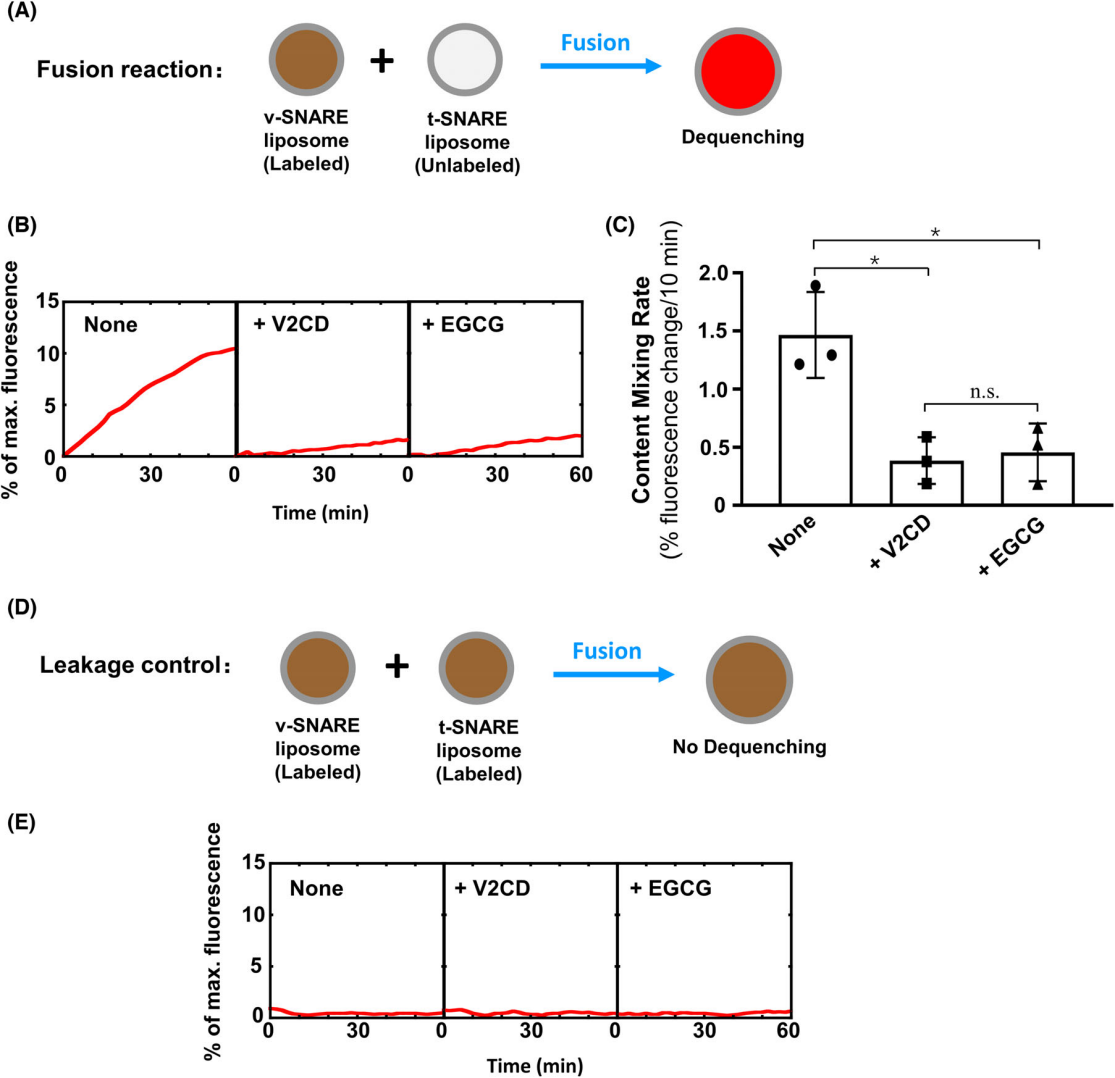
Epigallocatechin gallate inhibits the content mixing of SNARE‐mediated membrane fusion. (A) Diagram of the liposome‐liposome content mixing assay. The soluble dye sulforhodamine B (50 mm) was encapsulated in the v‐SNARE liposomes, in which its fluorescence was inhibited by self‐quenching. Fusion of the v‐SNARE liposomes with unlabeled t‐SNARE liposomes led to the dequenching of fluorescence. (B) Content mixing of the reconstituted fusion reactions. The v‐SNARE liposomes were directed to fuse with t‐SNARE liposomes in the absence or presence of 10 μm EGCG. Each fusion reaction contained 5 μm t‐SNAREs and 1.5 μm v‐SNARE. Data are presented as the fluorescence increase over time. In negative controls, 20 μm V2CD was added to the fusion reactions. (C) Initial content mixing rates of the fusion reactions shown in (B). Data are presented as a percentage of fluorescence change per 10 min. Error bars indicate the SD. Data are presented as the mean ± SD (*n* = 3 independent replicates). *P* values were calculated using ordinary one‐way ANOVA with Tukey's multiple comparisons test. n.s., *P* > 0.05; *P < 0.05. (D) Diagram of the leakage control reactions. Sulforhodamine B was included in both v‐ and t‐SNARE liposomes. (E) The leakage controls of the content mixing reactions. Increases in sulforhodamine B fluorescence were not observed, indicating that no detectable content leakage occurred during the fusion reactions.

We further examined how EGCG regulates SNARE‐driven content mixing [49, 50]. Sulforhodamine B was encapsulated in the VAMP2 liposomes in which its fluorescence was self‐quenched as a result of the high concentration. Fusion of the VAMP2 liposomes with unlabeled t‐SNARE liposomes led to the dilution of the dye and dequenching of fluorescence (Fig. 2A). Using this assay, we observed that the SNAREs drove a comparable level of content mixing. EGCG strongly blocked the SNARE‐mediated content mixing reaction similar to V2CD (Fig. 2B,C). In the leakage control reactions, the sulforhodamine B fluorescence was not increased, indicating that no content leakage occurred in the fusion reactions (Fig. 2D,E). Thus, EGCG has the ability to inhibit both the lipid and content mixing of SNARE liposomes.

**Fig. 3:**
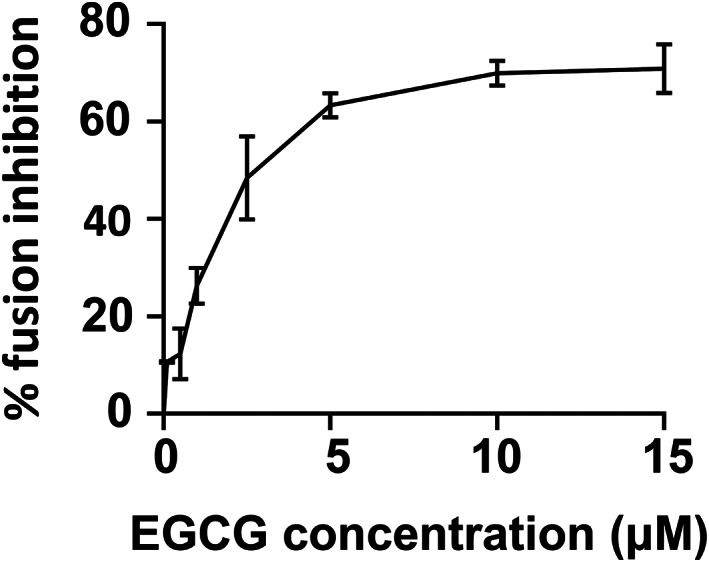
Dose dependence of EGCG activity in the SNARE‐dependent fusion reaction. EGCG was added to the reconstituted fusion reaction at the indicated concentrations. Each fusion reaction contained 5 μm t‐SNAREs and 1.5 μm v‐SNARE. Data are presented as a percentage of fusion inhibition at indicated concentration of EGCG. Error bars indicate the SD. Data are presented as the mean ± SD (*n* = 3 independent replicates).

We then examined the dose dependence of EGCG activity in the reconstituted SNARE‐dependent fusion reaction (Fig. 3). The maximum inhibition of fusion was reached with 10 μm EGCG (Fig. 3). Together, these data demonstrated that EGCG is an insulin secretory SNARE‐dependent membrane fusion inhibitor, which dose‐dependently blocks membrane fusion.

### EGCG inhibits membrane fusion by blocking the *trans*‐SNARE assembly

**Fig. 4:**
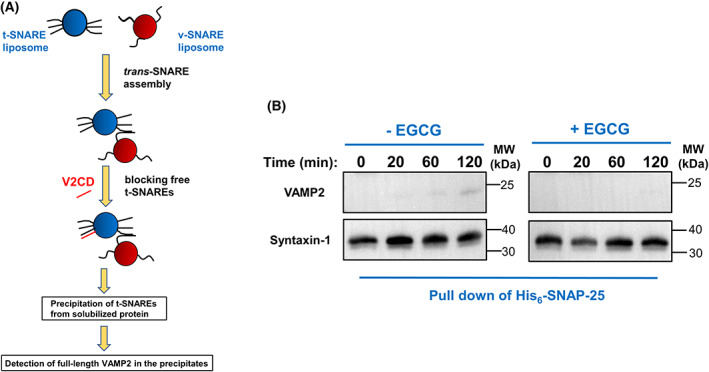
Epigallocatechin gallate blocks the formation of the *trans*‐SNARE complex. (A) Diagram of the *trans*‐SNARE formation assay. (B) Reconstituted t‐ and v‐SNARE liposomes were incubated at 4 °C for the indicated periods in the presence or absence of 10 μm EGCG before 10‐fold excess amount of inhibitory V2CD was added. The liposomes were subsequently solubilized and the t‐SNAREs were precipitated. The presence of FL VAMP2 in the precipitates was probed by western blotting, which was used as an indicator for the *trans*‐SNARE complex.

Membrane fusion required the energy provided by the zippering of the *trans*‐SNARE complex. We then tested how EGCG regulates the formation of the *trans*‐SNARE complex. At the low temperature of 4 °C, t‐ and v‐SNARE liposomes formed a *trans*‐SNARE complex resistant to V2CD but could not drive fusion [49, 50, 51]. We then added V2CD to block the unpaired t‐SNARE liposomes. After protein solubilization, the t‐SNAREs were pulled down by His6‐SNAP25. The full‐length VAMP2 was used to indicate the *trans*‐SNARE complex (Fig. 4A) [49, 50, 51]. Using this assay, we observed that EGCG strongly blocked the formation of the *trans*‐SNARE complex, which further inhibited the SNARE‐dependent membrane fusion (Fig. 4B).

### The inhibitory activity of EGCG in the fusion reaction is compatible with multiple v‐SNAREs

**Fig. 5:**
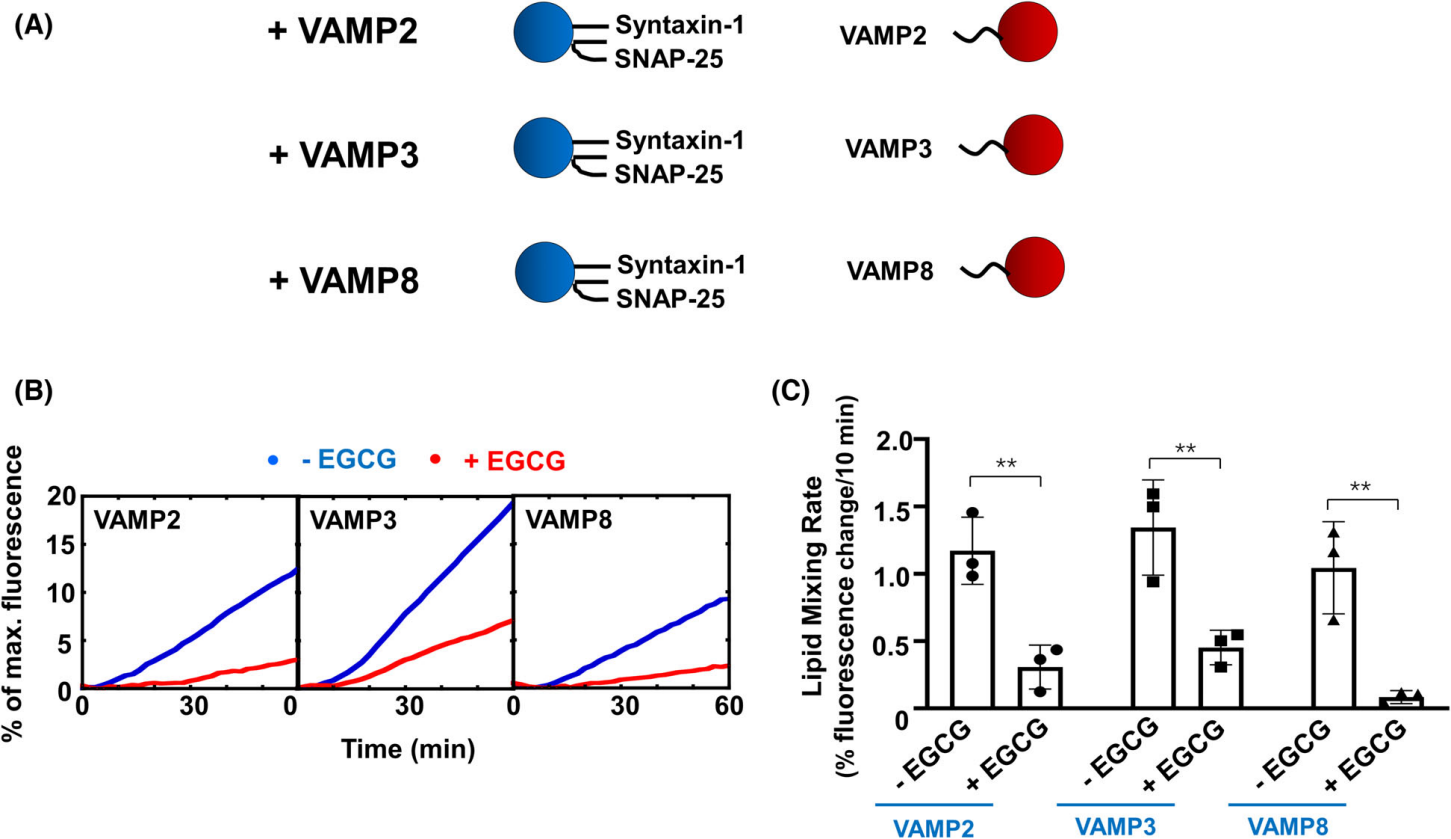
The inhibition of fusion by EGCG is compatible with multiple v‐SNAREs. (A) Illustrations of the liposome fusion pairs. (B) Lipid mixing of the reconstituted fusion reactions. Each fusion reaction contained 5 μm t‐SNAREs and 1.5 μm v‐SNARE. The fusion reactions were measured using a FRET‐based lipid mixing assay. (C) Initial lipid mixing rates of the liposome fusion reactions shown in (B). Data are presented as a percentage of fluorescence change per 10 min. Error bars indicate the SD. Data are presented as the mean ± SD (*n* = 3 independent replicates). *P* values were calculated using two‐way ANOVA with Tukey's multiple comparisons test. **P < 0.01.

Although VAMP2 is the primary v‐SNARE, VAMP3 and VAMP8 were also reported to participate in the regulation of insulin secretion [44, 45, 46]. They may serve as compensatory v‐SNAREs in insulin exocytosis. We then examined whether EGCG could inhibit the fusion reactions with VAMP3 or VAMP8. The t‐SNARE liposomes bearing syntaxin‐1 and SNAP‐25 were directed to fuse with liposomes reconstituted with VAMP2, VAMP3, or VAMP8 (Fig. 5A). Interestingly, EGCG dramatically reduced the fusion rate of all the fusion reactions (Fig. 5B,C). These data suggest that the suppression function of EGCG in the SNARE‐dependent fusion reaction is compatible with all the three v‐SNARE isoforms involved in insulin secretion.

### The inhibitory function of EGCG is dominant over the stimulatory activity of Syt7

Glucose‐stimulated insulin secretion is triggered by intracellular Ca^2+^, a second messenger. It has been established that Syt7 serves as the primary Ca^2+^ sensor during this process. When the blood glucose elevates, Syt7 recruits the insulin secretory granules to the PM and interacts with the SNARE proteins in a Ca^2+^‐dependent manner [25, 26, 27].

**Fig. 6:**
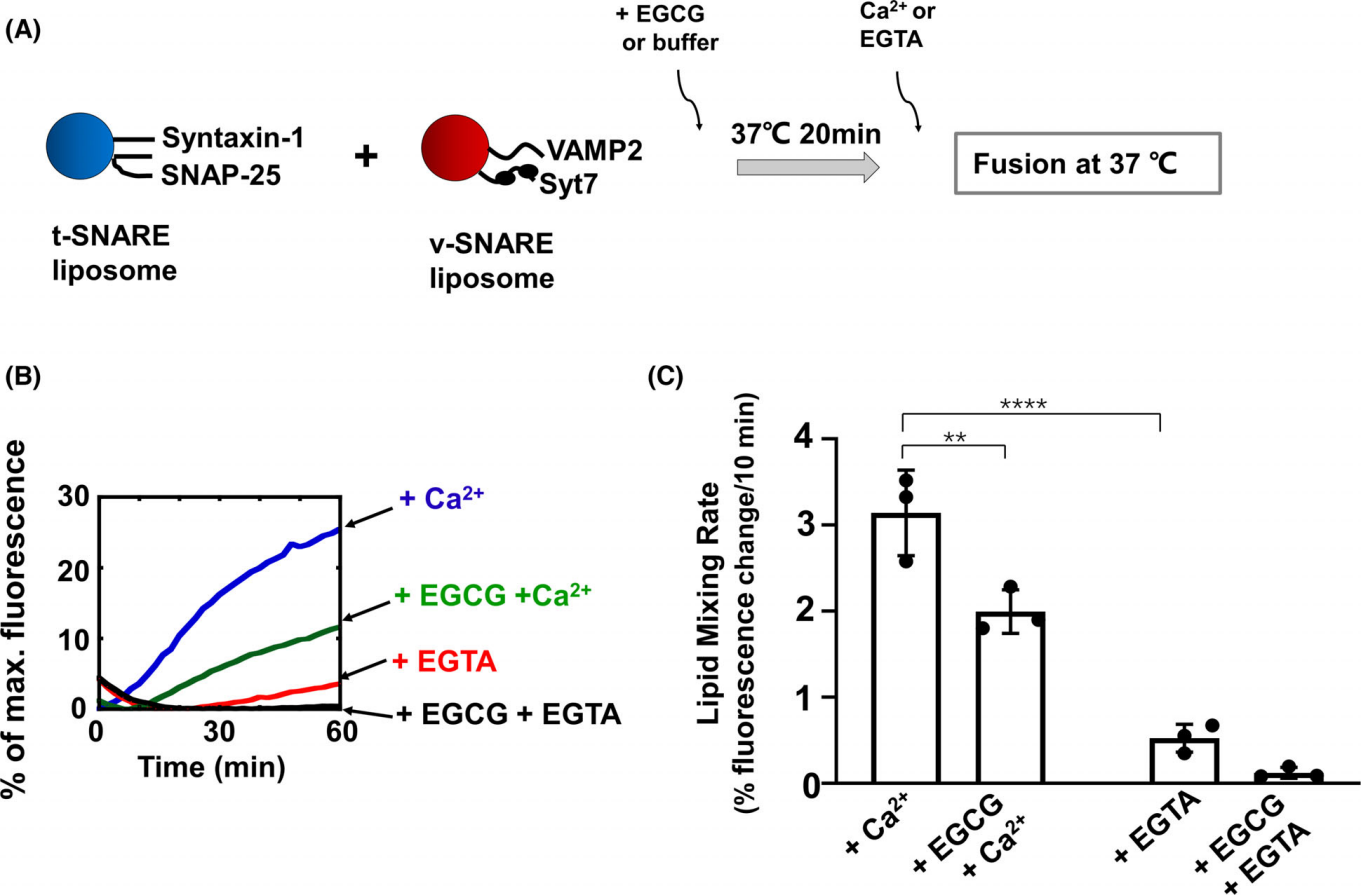
Epigallocatechin gallate inhibits insulin secretory SNARE‐dependent membrane fusion in the presence of Syt7 and Ca^2+^. (A) Illustrations of the liposome fusion procedures. The t‐SNARE liposomes containing syntaxin‐1 and SNAP‐25 were reconstituted using the lipid composition: 50% POPC, 20% POPE, 15% POPS, 10% cholesterol, 3% 1‐palmitoyl‐2‐oleoyl‐*sn*‐glycero‐3‐phosphoinositol (POPI) and 2% phosphatidylinositol‐4,5‐bisphosphate (PIP2). The v‐SNARE liposomes containing VAMP2 and Syt7 were prepared using the lipid composition: 47% POPC, 20% POPE, 15% POPS, 10% cholesterol, 5% POPI, 1.5% rhodamine‐DPPE and 1.5% NBD‐DPPE. The v‐ and t‐SNARE liposomes were mixed with 10 μm EGCG in the presence of 0.2 mm EGTA and 100 mg·mL^−1^ Ficoll 70. The samples were incubated at 37 °C for 20 min. Subsequently, 1 mm CaCl_2_ (or 1 mm EGTA) was added, and the fusion reactions were monitored for 60 min. (B) Lipid mixing of the reconstituted fusion reactions. The fusion reactions were measured by a FRET‐based lipid mixing assay. (C) Initial lipid mixing rates of the liposome fusion reactions shown in (B). Data are presented as a percentage of fluorescence change per 10 min. Error bars indicate the SD. Data are presented as the mean ± SD (*n* = 3 independent replicates). *P* values were calculated using two‐way ANOVA with Tukey's multiple comparisons test. **P < 0.01; ****P < 0.0001.

We then examined how EGCG influences membrane fusion in the presence of Syt7 and Ca^2+^. We expressed and purified recombinant full‐length Syt7 protein and reconstituted it to the v‐SNARE liposomes (Fig. 6A). In the lipid mixing assay, Ca^2+^ dramatically accelerated the Syt7‐ and SNARE‐mediated fusion kinetics (Fig. 6B,C). Strikingly, the fusion rates in the presence of Ca^2+^ were largely reduced by EGCG, suggesting that EGCG can arrest the fusion reaction in the presence of Syt7 and Ca^2+^ (Fig. 6B,C). Therefore, the inhibitory function of EGCG is dominant over the stimulatory activity of Syt7/Ca^2+^ in vesicle fusion, supporting that EGCG is a negative regulator of SNARE‐mediated membrane fusion in insulin secretion.

## Discussion

The incidence of insulin‐based metabolic diseases, such as type II diabetes and hyperinsulinemia, has increased rapidly in the past decades [52]. As a result of minor side effects and healthy resources, dietary polyphenol drugs are currently attracting special attention [36]. EGCG is the most abundant polyphenol in green tea, which can reduce the risk of diabetes and other metabolic complications [41, 42, 43]. Insulin, the critical blood glucose‐lowering hormone, plays a central role in glucose metabolism. How EGCG regulates insulin secretion remains poorly understood.

Because of the complexity of the intracellular environments, it is challenging to examine or screen molecules targeting exocytosis *in vivo*. In the present study, we aimed to dissect the insulin secretion pathway by reconstituting insulin secretory vesicle fusion *in vitro* using purified components. The SNARE and Syt7 protein composition and topology can be precisely controlled in this defined fusion system. Regulators or molecules can be directly added without the complications of other molecules naturally present in the cell, allowing their kinetic effects on fusion to be causally established [48].

Based on this *in vitro* reconstituted system, we explored the role of EGCG on SNARE‐dependent insulin secretion. We found that EGCG can strongly suppress the dynamics of SNARE‐mediated lipid mixing and content mixing. The dose‐dependent inhibitory effect suggested that the fusion inhibition is solely caused by EGCG. How does EGCG arrest the membrane fusion? Using our previously developed *trans*‐SNARE assembly assay, we demonstrated that EGCG efficiently blocks the zippering of the *trans*‐SNARE complex, which is formed by t‐ and v‐SNAREs from the opposed membrane. Hence, EGCG disrupts the *trans*‐SNARE assembly to inhibit the SNARE‐dependent membrane fusion.

Our data showed the inhibitory activity of EGCG is compatible with a variety of v‐SNARE isoforms, including VAMP2, VAMP3 and VAMP8, consistent with the physiological observation of these v‐SNAREs supporting insulin secretion *in vivo* [53, 54]. Because insulin secretion is simulated under the regulation of Ca^2+^, we then investigated how EGCG affects membrane fusion in the presence of Syt7 and Ca^2+^. Interestingly, EGCG apparently inhibits the fusion reactions under the stimulation of Syt7/Ca^2+^, consistent with our conclusion that EGCG inhibits insulin secretory vesicle fusion through the step of *trans*‐SNARE assembly.

Multiple studies demonstrated the onset of hyperinsulinemia in patients affected by metabolic syndrome and the role of GTE in improving obesity, diabetes and other metabolic syndromes [55, 56]. In this work, we characterized a new target of EGCG, one of the main polyphenols in GTE, by presenting how EGCG regulates SNARE‐driven insulin secretion. On this basis, supplementing GTE or EGCG as an adjuvant in the diet is a helpful nutritional strategy in caring for insulin‐dependent metabolic disorders, including hyperinsulinemia.

## Materials and methods

### Protein expression and purification

Recombinant t‐ and v‐SNARE proteins were expressed in *Escherichia coli* strain BL21(DE3) and purified by nickel affinity chromatography, using a previously established procedure [49, 50, 57]. The t‐SNARE complex was composed of untagged rat syntaxin‐1 and mouse SNAP‐25 with an N‐terminal His_6_ tag. Recombinant v‐SNARE proteins had no extra residues after the tags were proteolytically removed by SUMO protease [49, 50, 57]. The full‐length gene for Syt7 was cloned into a pET28a‐based SUMO vector. Purified His6‐SUMO‐Syt7 fusion proteins were digested by SUMO proteases to remove the extra tags. SNAREs and Syt7 were stored in a buffer containing 25 mm Hepes (pH 7.4), 400 mm KCl, 1% *n*‐octyl‐β‐d‐glucoside, 10% glycerol and 1 mm dithiothreitol.

### Reconstitution of proteoliposomes

All of the lipids used in the present study were obtained from Avanti Polar Lipids Inc. (Alabaster, AL, USA). For t‐SNARE reconstitution, 1‐palmitoyl‐2‐oleoyl‐*sn*‐glycero‐3‐phosphocholine (POPC), 1‐palmitoyl‐2‐oleoyl‐*sn*‐glycero‐3‐phosphoethanolamine (POPE), 1‐palmitoyl‐2‐oleoyl‐*sn*‐glycero‐3‐phosphoserine (POPS) and cholesterol were mixed in a molar ratio of 60 : 20 : 10 : 10. For v‐SNARE reconstitution, POPC, POPE, POPS, cholesterol, *N*‐(7‐nitro‐2,1,3‐benzoxadiazole‐4‐yl)‐1,2‐dipalmitoyl phosphatidylethanolamine (NBD‐DPPE) and *N*‐(Lissamine rhodamine B sulfonyl)‐DPPE (rhodamine‐DPPE) were mixed at a molar ratio of 60 : 17 : 10 : 10 : 1.5 : 1.5. SNARE proteoliposomes were prepared by detergent dilution and isolated on a Nycodenz (Axis‐Shield, Dundee, UK) density gradient [51, 58]. Complete detergent removal was achieved by overnight dialysis of the samples in Novagen dialysis tubes against the reconstitution buffer [25 mm Hepes (pH 7.4), 100 mm KCl, 10% (vol/vol) glycerol and 1 mm dithiothreitol]. To prepare sulforhodamine‐loaded liposomes, SNARE liposomes were reconstituted in the presence of 50 mm sulforhodamine B (Sigma, St Louis, MO, USA). Free dye was removed by overnight dialysis, followed by liposome flotation on a Nycodenz gradient. The protein/lipid ratio was 1 : 200 for v‐SNAREs and 1 : 500 for t‐SNARE liposomes.

### Preparation of EGCG solution

Epigallocatechin gallate powder was dissolved in ddH_2_O to obtain a 20 mm stock solution. For each experiment, EGCG was diluted to the required concentration using reconstitution buffer.

### Lipid mixing assay

A standard lipid mixing reaction contained 5 μm t‐SNAREs and 1.5 μm v‐SNARE. v‐SNARE liposomes labeled with NBD and rhodamine were mixed with t‐SNARE liposomes in the presence or absence of EGCG to initiate fusion. The fusion reactions were conducted in a 96‐well microplate at 37 °C ^[^ ^14^, ^48^ ^]^. NBD fluorescence (excitation: 460 nm; emission: 538 nm) was measured every 2 min in a Synergy HT microplate reader (BioTek, Winooski, VT, USA). At the end of the reaction, 10 μL of 10% CHAPSO was added to each sample. Fusion data were presented as the percentage of maximum fluorescence change. Full accounting of statistical significance was included for each dataset based on at least three independent experiments.

### Content mixing assay

In the content mixing assays, unlabeled t‐SNARE liposomes were directed to fuse with sulforhodamine B‐loaded v‐SNARE liposomes in which sulforhodamine B fluorescence was inhibited by self‐quenching. The fusion of the liposomes led to the mixing of their contents and the dequenching of sulforhodamine B fluorescence [49, 50, 51]. The sulforhodamine B fluorescence (excitation: 565 nm; emission: 585 nm) was measured every 2 min. At the end of the reaction, 10 μL of 10% CHAPSO was added to each sample. Fusion data were presented as the percentage of maximum fluorescence change. Full accounting of statistical significance was included for each dataset based on at least three independent experiments.

### *Trans*‐SNARE assembly assay

The *trans*‐SNARE assembly assay was performed as described previously [49, 50, 51]. Reconstituted t‐ and v‐SNARE liposomes were incubated at 4 °C for the indicated periods in the presence or absence of EGCG before a 10‐fold excess amount of inhibitory GST‐tagged V2CD was added to block unpaired t‐SNAREs. The liposomes were subsequently solubilized by 1% CHAPSO and the t‐SNAREs were precipitated using nickel sepharose beads. The presence of full‐length VAMP2 in the precipitates was probed by immunoblotting, which was used as an indicator for the *trans*‐SNARE complex formed between liposomes. Syntaxin‐1 probed by immunoblotting was used as an indicator of t‐SNAREs in the precipitates.

### Statistical analysis

All data were presented as the mean ± SD and were analyzed using prism, version 8.0.2 (GraphPad Software Inc., San Diego, CA, USA). Statistical significance was calculated using one‐way analysis of variance (ANOVA) or two‐way ANOVA. *P* < 0.05 was considered statistically significant.

## Conflict of interest

The authors declare no conflict of interest.

## Author contributions

YL and HY conceived the project. MZ, HX and YJ performed the experiments. MZ, HX and YJ analyzed the data. MZ, HY and YL wrote the manuscript with input from all authors.

## Data Availability

The data that support the findings of the present study are available from the corresponding author upon reasonable request.
